# Heat Modulation of Intrinsic MR Contrasts for Tumor Characterization

**DOI:** 10.3390/cancers14020405

**Published:** 2022-01-14

**Authors:** Matthew Tarasek, Oguz Akin, Jeannette Roberts, Thomas Foo, Desmond Yeo

**Affiliations:** 1 GE Global Research, Niskayuna, NY 12309, USA; rroberts@ge.com (J.R.); Thomas.Foo@ge.com (T.F.); yeot@ge.com (D.Y.); 2 Memorial Sloan-Kettering Cancer Center, Department of Radiology, New York, NY 10065, USA; akino@mskcc.org

**Keywords:** tissue characterization, MRI, thermal sensitivity, in vivo

## Abstract

**Simple Summary:**

Multi-parametric magnetic resonance imaging (MRI) is a paradigm that combines several MR imaging contrast types to provide added layers of information for the characterization of tissue types, including benign and malignant tumors. The approach detailed in this manuscript evaluates heat-induced changes in intrinsic MRI contrast types in vivo for tumor characterization. Specifically, the quantitative longitudinal relaxation time (T_1_), transverse relaxation time (T_2_), water proton chemical shift (CS), and apparent diffusion coefficient (ADC) were measured at various temperatures for benign and malignant tumors in rats. Results indicate that heat-induced changes in these intrinsic contrast types can potentially improve MR imaging visualization and characterization of tumor tissue. The approach detailed here may have a strong impact on real-time interventional procedures where tumor boundaries need to be accurately delineated to maximize positive therapeutic response for MRI-guided focal therapy.

**Abstract:**

(1) Background: The longitudinal relaxation time (T_1_), transverse relaxation time (T_2_), water proton chemical shift (CS), and apparent diffusion coefficient (ADC) are MR quantities that change with temperature. In this work, we investigate heat-induced intrinsic MR contrast types to add salient information to conventional MR imaging to improve tumor characterization. (2) Methods: Imaging tests were performed in vivo using different rat tumor models. The rats were cooled/heated to steady-state temperatures from 26–36 °C and quantitative measurements of T_1_, T_2_, and ADC were obtained. Temperature maps were measured using the proton resonance frequency shift (PRFS) method during the heating and cooling cycles. (3) Results: All tissue samples show repeatable relaxation parameter measurement over a range of 26–36 °C. Most notably, we observed a more than 3.3% change in T_1_/°C in breast adenocarcinoma tumors compared to a 1% change in benign breast fibroadenoma lesions. In addition, we note distinct values of T_2_/°C change for rat prostate carcinoma cells compared to benign tissue. (4) Conclusion: These findings suggest the possibility of improving MR imaging visualization and characterization of tissue with heat-induced contrast types. Specifically, these results suggest that the temporal thermal responses of heat-sensitive MR imaging contrast mechanisms in different tissue types contain information for improved (i) characterization of tumor/tissue boundaries for diagnostic and therapy purposes, and (ii) characterization of salient behavior of tissues, e.g., malignant versus benign tumors.

## 1. Introduction

The clinical applications of magnetic resonance (MR) imaging in oncology are rapidly evolving from subjective and interpretive diagnostic tests based on tissue morphology to more quantitative approaches that probe tissue biology. The best possible characterization of cancer in MR imaging is currently achieved by a multi-parametric approach that supplements conventional MRI with additional functional MRI techniques [1,2,3,4]. These techniques provide added layers of information on features such as tumor metabolism, cellular microenvironment, and tumor vascularity [2,4,5,6,7]. Despite ongoing research into multi-parametric MRI [8,9,10,11], there are still limitations in detecting and delineating early-stage cancer lesions when they are curable [4]. MR imaging provides the best spatial resolution and anatomical soft tissue contrast, but there is still a need to develop novel MR imaging approaches to improve tissue characterization, reduce unwanted biopsies, and provide additional information to guide cancer therapy treatments. The lack of multi-parametric MR datasets is a major clinical impediment in cancer screening and therapy planning, even in the most common cancer types.

In this work, we evaluate a novel approach to characterize tissues using the thermal responses of heat-sensitive MR quantities. We provide an initial dataset to assess how thermally sensitive intrinsic MR contrast types may differentiate certain tissue types, with the ultimate goal of improving tumor visualization and characterization. In general, tumors have different mechanical, water diffusion, thermal, and perfusion properties from normal tissue [2,12]. These can produce different thermal responses in proton resonance frequency shift (PRFS)-based MR thermometry, T_1_, T_2_, and diffusion-weighted imaging [13,14,15,16]. It is challenging to use all these descriptors in a multivariate MR thermal contrast approach because the way they change with temperature is very tissue dependent. Previous work detailing the temperature dependence of various MR quantities is mostly limited to ex vivo analysis [17,18,19,20], although some in vivo testing has been done using T_2_ for the thermometry of adipose tissue in humans [21]. To the best of our knowledge, there is very limited work detailing how these thermal descriptors change with temperature in vivo, especially for different tumor types.

In this report, we present preclinical in vivo animal data that quantify how T_1_, T_2_, PRFS, and ADC change with temperature in breast carcinoma, breast fibroadenoma, and prostate carcinoma. The results suggest that these thermal response characteristics may be useful as an additional imaging biomarker in a multiparametric framework for improved cancer diagnosis and treatment monitoring. To that end, we envision a method that consists of (i) a thermal applicator, (ii) an optimized set of MR protocols that acquires T_1_, T_2_, and diffusion-weighted maps at various temperatures, and (iii) a classification module that interprets the acquired data for cancer diagnosis and treatment response.

## 2. Materials and Methods

The procedure to perform in vivo temperature change experiments involved (1) administering anesthesia and positioning the rat inside the coil, (2) placing the rectal thermometer, tumor surface thermometer, insulation, and heat source, (3) achieving a “low temperature” 26-degree Celsius steady-state rectal (body) temperature for 10 min before quantitative scanning, and (4) warming the rat to a “high temperature” 36-degree Celsius temperature for final quantitative scanning. A rectal probe and a fiber optic temperature probe placed on the shaved tumor surface were used to verify temperatures for the body and tumor, respectively. MR thermometry (MRT) was also used to measure temperature changes during periods of warming. Localizer scans were performed as the rat temperature stabilized during Step 3 and tumor locations were determined with the help of a knowledgeable biologist. Axial slice selection was performed to cover the entire tumor volume. In general, two to four slices gave full tumor coverage. Once the rat reached steady-state temperature (i.e., <Δ0.1 °C/min), quantitative measurements of T_1_, T_2_, and ADC were obtained. In total, 12 rats were scanned with four rats for each tumor type.

Two tumor cell lines were used in this study. MAT B III cells, rat breast adenocarcinoma (ATCC^®^ CRL-1666^TM^), were cultured in McCoy’s 5A media supplemented with 10% FBS. MatLyLuB2 cells, rat prostate carcinoma (ATCC^®^ CRL-2376^TM^), were cultured in RPMI 1640 media supplemented with 10% FBS. The MAT B III cells were authenticated and tested at ATCC for post freeze viability, morphology, mycoplasma contamination (Hoechst DNA Stain and Direct Culture Methods), post freeze cell growth, interspecies determination (Isoenzyme Analysis), and bacterial and fungal contamination. The MatLyLuB2 cells were authenticated and tested at ATCC for post freeze viability, growth properties, morphology, mycoplasma contamination (Hoechst DNA Stain and Direct Culture Methods), interspecies determination (COI assay), and sterility test (BacT/ALERT 3d: iAST bottle aerobic and anaerobic at 32 °C). Cells underwent Radil testing in-house using the IMPACT V PCR profile method for H1, KRV, LCMV, MAD, *Mycoplasma pulmonis*, *Mycoplasma* sp., PVM, rCMV, RCV/SDAV, REO3, RMV, RPV, RTV, Sendai, Seoul. All test results were negative. No other internal authentication testing was done.

The experimental protocol was approved by the IACUC at the General Electric Global Research Center (Niskayuna, NY, USA), which is an AAALAC and OLAW accredited facility. Animals were housed in standard caging, were provided food and water ad libitum and were maintained on a 12-h/12-h light/dark cycle. In this study, we used 8–12 week female Fischer 344 rats (MAT B III cell line), 8–12 week male Copenhagen rats (MatLyLuB2 cell line), and 18–24 month female Sprague Dawley rats (spontaneous mammary tumors). Rats received a subcutaneous injection of 5–10 × 10^6^ cells suspended in complete media to their left flanks. Older Sprague Dawley rats were observed until spontaneous palpable growths were detected. When the tumors reached an optimal size, the rats were imaged in the MRI scanner. During imaging the rat was anesthetized using 1–3% isoflurane.

After imaging and heating experiments, histopathology was performed on the tumor tissue. Tumors were collected and fixed in 10% formalin for 24 h. Tissues were processed using a Tissue Tek VIP tissue processor, embedded in paraffin and sectioned into 5 μm sections. Sections were stained using a standard H&E staining protocol, and the whole tissue section was imaged in 20× magnification using a VS120 whole slide scanner (Olympus, Center Valley, PA, USA). This technique is considered the gold standard for analyzing histology slides and was essential in determining the diagnosis for the spontaneous mammary tumors. An in-house pathologist was consulted to evaluate the H&E slides and provide a diagnosis.

Rats with breast adenocarcinoma (MAT B III), benign breast fibroadenoma, and prostate carcinoma (MatLyLuB2) were imaged when the tumors reached a diameter of 2–3 cm. Imaging tests were performed in vivo, and quantitative metrics were calculated for all tumor tissue and muscle tissue near the tumors. Rats were anaesthetized using isoflurane and placed into a rat-sized transmit/receive quadrature Litz rat coil (Doty Scientific). Imaging was performed on a 3T GE MR750 scanner (GE Healthcare, Waukesha, WI, USA). The core body temperature of the rat was monitored rectally by a calibrated fiber-optic thermometer (SA Instruments, Stony Brook, NY, USA) along with a fiberoptic thermometer attached to the shaved (hair-free) surface of the tumor growth (fluoroptic thermometry system Luxtron M3300, Lumasense Technologies, Santa Clara, CA, USA). Rats were heated by either (i) hot water flow through tube lines, (ii) an MR-compatible resistive heat pad (CWE, Inc. Ardmore, PA, USA) that replaced the water lines, and/or (iii) an MR compatible small animal heating system (SA Instruments) which contained a hot-air blower controlled by the temperature probe sensor.

**Figure 1:**
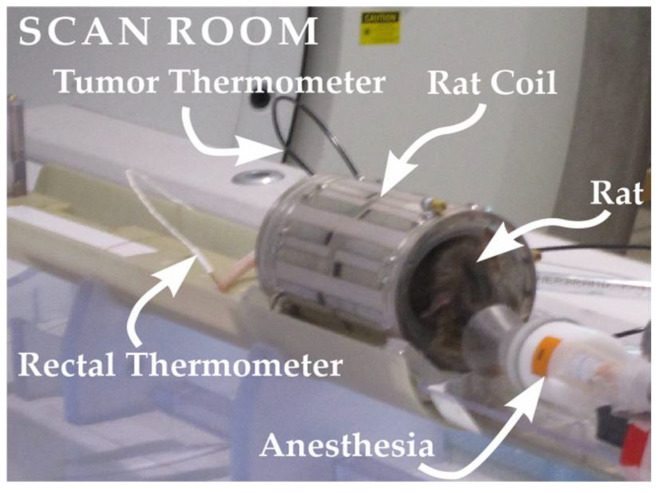
Setup used for imaging/heating experiments in rats (shown without oil vials, or insulation). Rats were administered an isoflurane anesthesia and placed into a rat-sized transmit/receive MRI coil. The core body temperature of the rat was monitored rectally by a calibrated fiber-optic thermometer. The rats were stabilized at temperatures for 10 min to achieve a steady state before imaging experiments began.

The system was thermally isolated, and the rats were stabilized at temperatures for 10 min to ensure a steady state before imaging experiments began. Figure 1 shows the general setup for experiments (shown without insulation material or oil vials). The core body temperature was maintained at temperatures of 26 and 36 °C. The “low temperature” of 26 °C was achieved by allowing free breathing of room temp air (~21 °C) under isoflurane anesthetic without any heating. The rat body temperature dropped naturally to 26 °C at which point it was stabilized with active heating. Two cycles of cooling and warming were performed per rat. Data were acquired for four rats per tumor type.

**Figure 2:**
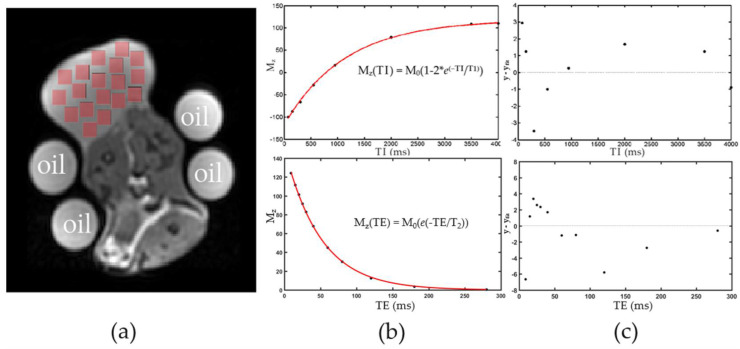
Example of T_1_ and T_2_ curve fit and analysis. (**a**) Example image through the center mass of the tumor. Signal intensity in each square ROI was averaged and contributes to one point in a T_1_ or T_2_ plot. (**b**) T_1_ plot (top) and T_2_ plot (bottom) was approximated as a single exponential recovery/decay. (**c**) Residuals from the fits in (**b**).

T_1_ datasets were acquired using a 2D axial inversion recovery (IR) sequence at the following TI values: 4000, 3500, 2000, 950, 550, 350, 150, and 75, with all times in ms. Other parameters included flip angle (θ) = 180/90° set with proper transmit gain setting (TG) of RF amplifier, TR = 6000 ms, TE = 4 ms, FoV = (13 × 6.5) cm^2^, matrix 128 × 128, NEX = 1, 5 slices, 3 mm slice thickness. T_1_-IR signal intensity (S) was fit to (1)

(1) $$S\left(\tau \right)=S_{0}\left(1-2e^{-TI/T_{1}}\right),$$

over either a selected region of interest (ROI) or per pixel, where (*TI*) is the inversion time. T_2_ datasets were acquired using a 2D axial spin echo (SE) sequence with the following TE values: 280, 180, 120, 80, 60, 40, 30, 25, 20, 15, and 4 ms, θ = 90/180° (set with proper TG), and same parameters as the IR experiments. T_2_-SE signal intensity (S) was fit to (2)

(2) $$S\left(\tau \right)=S_{0}e^{-TE/T_{2}}$$

over either a selected ROI or per pixel where (*TE*) is the echo time. Figure 2a–c depicts the general data-processing procedure performed per slice for relaxation parameter measurements. Figure 2a illustrates single slice ROI selections for data fitting. Each ROI averages the signal intensity of its voxels and represents one point on a plot like Figure 2b. Here, 18 regions of interest, each consisting of 38 × 38 voxels, were taken at each inversion time (inversion time = 150 ms in Figure 2a) and T_1_ plots were made for each ROI. This procedure was repeated for a 1–5 slice acquisition giving typically 20–80 T_1_ measurements (depending on the number of ROIs taken from muscle/tumor regions) to be used for statistics calculations.

ADC datasets were acquired from diffusion-weighted images (DWI) using a 2D axial spin echo (SE) sequence with echo-planar imaging (EPI) readout. Other parameters included θ = 90°/180° (set with proper TG), TR = 6000 ms, FoV = (13 × 6.5) cm^2^, matrix size = (128 × 128), 5 slices, slice thickness = 3 mm, b-values of 300, 600, 900, 1200 s/mm^2^, and NEX = 8 for each b-value. Post processing was performed on the scanner console where the ADC of each pixel was computed from a fit of (3)

(3) $$S\left(b\right)=S_{0}e^{-bADC}$$

where *S*(*b*) is the voxel signal intensity at some b-value and *S*_0_ is the signal intensity at a b-value of 0.Γ.

The change in the set of quantitative thermal MR quantities (Γ = T_1_, T_2_, ADC) were measured and described with respect to a unit change in temperature (%ΔΓ/°C). Calculations were made using the Equation (4)

(4) $$\%\Delta \Gamma /{}^{\circ}C=\left(\left(\Gamma_{\mathrm{highT}}-\Gamma_{\mathrm{lowT}}\right)/\Gamma_{\mathrm{lowT}}\right)/\Delta {}^{\circ}C\times 100\%,$$

where Γ_highT_ is the high temperature (36 °C) measure of ΔΓ and Γ_lowT_ is the low temperature measurement at 26 °C. All quantitative imaging metrics were compared using a standard Student’s *t*-test for two samples. We rejected the null hypothesis (i.e., no difference between muscle and tumor tissue means, or respective rates of change) with a 5% type I error probability (*p* < 0.05).

A 3-echo spoiled gradient echo (SPGR) imaging sequence was used to perform MR thermometry (MRT) during periods of temperature change. In this work MRT was used as a complementary method to monitor internal tumor and muscle temperature changes during periods of warming. MRT data were used to confirm internal tissue temperature change for comparison with the fiber optic probe measurements. Sequence parameters included TE_1_ = 14.9 ms, TE_2_ = 17.3 ms, TE_3_ = 19.7 ms, TR = 110 ms, θ = 29°, FoV = (13 × 13) cm^2^, matrix size = (128 × 128), 3 slice acquisition, slice thickness = 7 mm. Fat-referenced PRFS maps [22,23,24,25] are computed as (5)

(5) $$\Delta T\left(x,y\right)=\frac{\left(x,y\right)-\Delta \phi_{f}\left(x,y\right)}{\gamma \alpha B_{0}TE}$$

where $\Delta \phi_{f}\left(x,y\right)$ represents the non-temperature dependent component of the PRF phase shift during heating experiments, assumed to be dominated by static B_0_ drift. The water-based phase difference maps are corrected by the phase difference maps of the fat images as any fat phase change is assumed to be caused by time-varying phase disturbances [26]. To avoid any confounding results from in vivo fat susceptibility changes with increasing temperature, unheated external oil vials were used as the correction region. Corrected PRFS MRT techniques used here have been thoroughly validated to produce MRT accuracy and precision values of <0.5 °C in phantoms [27]. For these experiments, the PRFS thermal coefficient for water-based tissues (α = −0.01 ppm/°C) was used. Rats were imaged at the magnet isocenter.

## 3. Results

### 3.1. MR Imaging Experiments with Temperature Modulation

**Figure 3:**
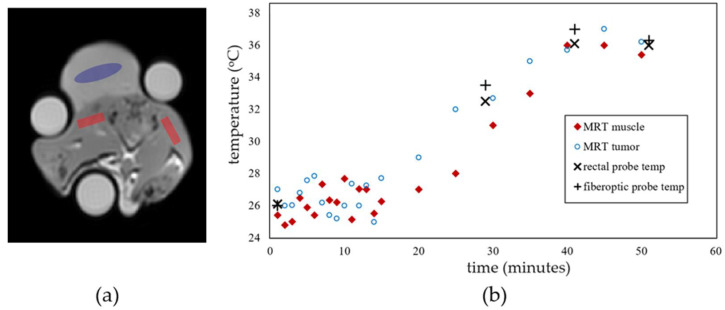
In vivo MR thermometry data and temperature probe data. (**a**) Axial slice through the center of a tumor grown on a rat flank. The elliptical ROI averages the MRT data in the tumor region, while the rectangular ROIs average MRT data in the muscle region. (**b**) Temperature change plots during warming cycle for muscle (diamond markers) and tumor (round markers) tissues. Plot also indicates rectal (body) and tumor temperature probe readings at various timepoints.

MR thermometry and temperature probe data were used to ensure that steady-state temperatures were achieved before quantitative parameter measurements were acquired. Figure 3a, b depicts warming data plots with MRT and temperature probe data for tumor tissue (blue oval ROIs) and surrounding muscle tissues (red rectangle ROIs). Muscle ROIs were selected to minimize fat content. The oil vials seen in the axial images were used for B_0_ drift correction in the PRFS measurements. External oil phantoms were also used to verify reproducibility in T_1_/T_2_ measurement at high and low temperatures [28]. All oil T_1_/T_2_ measurements were within 2.5% during the 2-h scan sessions. Similar plots to Figure 3b were observed for all rats imaged and provide a confirmation of temperature change and stabilization for quantitative imaging. MRT measurements were always within 1 °C of fiber optic probe measurements. All rats were heated from an initial temperature of 26 °C as discussed in the Materials and Methods section.

**Figure 4:**
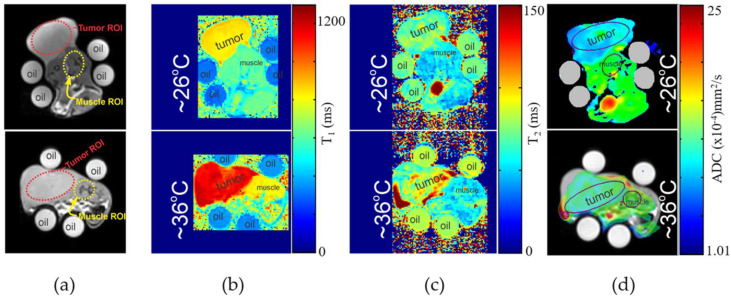
(**a**) Reference axial images of the same rat (top and bottom) with MAT B III tumor. Top images in (**b**–**d**) are quantitative T_1_, T_2_, and ADC maps, respectively, at 26 °C. Bottom images in (**b**–**d**) are quantitative T_1_, T_2_, and ADC maps, respectively, at 36 °C. General locations of the tumor and muscle regions are indicated on the images.

**Figure 5:**
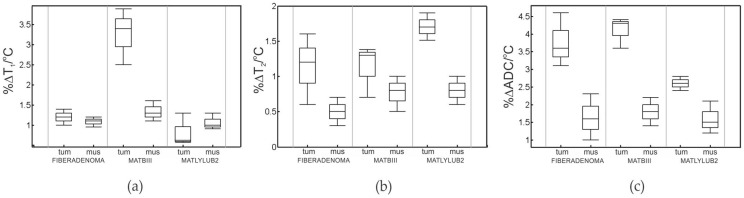
Box plots of MR thermal contrast change with temperature: (**a**) %ΔT_1_/°C, (**b**) %ΔT_2_/°C, and (**c**) %ΔADC/°C. Columns represent benign breast, malignant breast (MAT B III), and malignant prostate (MatLyLuB2), respectively (labeled “tum”), and muscle regions that were selected near those respective tumors (labelled “mus”). Each point plotted here represents 4 measurements for each tumor type or its surrounding muscle tissue.

For all tumor models analyzed in this work, we found a statistically significant difference (*p* < 0.05) between one or more delta-quantitative contrast measurements (%ΔΓ/°C) for all tumor/muscle pairs. Figure 4a–d presents an example of low-temperature and high-temperature quantitative image parameter maps for reconstructed T_1_, T_2_, and ADC images of a MAT B III tumor. Here, MRI data were fitted per pixel to obtain the quantitative maps. In all experiments the rat required some repositioning to check temperature probe placements. An example of this repositioning can be seen when comparing positional differences in Figure 4 top and bottom. Overall, the slices selected for the imaging volume were very similar as this was determined by the center mass of the xenograft tumor in localizer scans. Unfortunately, due to movement a direct mapping of parameter change could not be achieved. Table 1 shows the fit quantities (mean and standard deviation) for each tumor/muscle contrast type, and their respective %ΔΓ/°C. Most notably, we found significant difference in %ΔT_1_/°C and %ΔADC/°C for MAT B III tumor compared to its surrounding muscle, along with a significant difference in %ΔT_2_/°C and %ΔADC/°C for MatLyLuB2 compared to its surrounding muscle tissue. Blue bolded values in Table 1 indicate the statistically significant differences for all measured %Δ°C (t-test result *p*-value < 0.05). Box plots of highlighted values from Table 1 are presented in Figure 5. Each point plotted here represents four measurements for each tumor type or its surrounding muscle tissue.

### 3.2. Tumor Histopathology

Histopathology provided a ground truth reference to determine a true positive result for malignant or benign tissue. A histopathology report of each tumor used in experiments was made to determine the general tissue morphology of the tumors as described in section II.A. H&E staining of the MAT B III and MatLyLuB2 tumors indicates that these tumors are indeed malignant breast and prostate carcinoma, respectively. When evaluating the H&E staining of the spontaneous mammary tumors, all were classified as benign fibroadenomas.

## 4. Discussion

This study investigated how quickly quantitative MR quantities (T_1_, T_2_, PRFS and ADC) change with moderate temperature rise in vivo. This is in contrast to previous studies that characterized similar quantities in heated ex vivo tissue at temperatures above 37 °C [17,18,19,20]. Our results show that the tissue-dependent differences in how fast quantitative MRI quantities change as temperatures increase may potentially be used as biomarkers for tumor characterization. When compared to healthy muscle tissue, we observed statistically significant differences (*t*-test *p*-value < 0.05) in %ΔT_1_/°C and %ΔADC/°C in breast carcinoma (MAT B III), and %ΔT_2_/°C and %ΔADC/°C in prostate carcinoma (MatLyLuB2).

Although the exact biological mechanism for this thermal biomarker is still currently under investigation, it may be explained by a change in water molecules bound in different vascular and tissue compartmental structures of the tumors. A basic assumption is that upon heating, the proton correlation times change as water changes from a “bound state” to a “free state” inside the complicated vascular network, leading to the observed changes in T_1_, T_2_, and ADC. We do note however that observed changes more likely manifest from a very complicated combination of cellular mobility of water, vascular changes, macromolecule environment (itself a complex milieu of pH-dependent metabolites and proteins), and possible changes in tissue oxygenation. Future studies are needed to further elucidate the mechanism.

Several techniques can be used to induce temperature changes in vivo, including superficial and deep radiofrequency hyperthermia, MR-guided high-intensity focused ultrasound, microwave and laser ablation devices, and hypothermic methods [29,30,31,32,33,34]. Given the data summarized in Table 1 and Figure 5, statistically significant changes in selected quantitative MRI contrast types were observed in malignant tissue versus surrounding normal tissue over the range of temperatures studied here (26–36 °C). This suggests that the proposed tumor characterization approach may utilize sub-lethal hyperthermic temperatures (i.e., cumulative equivalent minutes at 43 °C < that for cell death in the anatomical regions of interest) instead of higher temperatures that may be cytotoxic within a shorter period of time. This technique can be particularly useful in circumstances where intrinsic relaxation quantities or diffusion imaging contrast at ambient temperature are not noticeably different between malignant and normal tissues. As an additional advantage, this technique does not require the administration of any exogenous contrast agent (Gd/Mn/^13^C based drug [35,36]). Any implementation of this technique will, however, depend on the availability of fast and accurate T_1_, T_2_, and ADC mapping techniques.

While the results of this study are promising, we acknowledge that the study can be further improved in future work to address some limitations. These limitations include the following:

(i) measurements were obtained at temperatures less than or equal to 36 °C;
(ii) accuracy of tumor temperature can be improved as temperature probe was placed just below the surface of the tumor instead of within the center of the tumor mass;
(iii) the relatively small number of in vivo samples (*n* = 4) for each tumor type;
(iv) the use of a xenograft (versus orthotopic) tumor model.

In (i), temperatures less than or equal to 36 °C were chosen to avoid the risks of adverse animal reactions potentially caused by whole-body hyperthermic temperatures for prolonged periods of time. The second limitation (ii) is salient because the temperatures of the tumor and surrounding muscle tissues are assumed to be equal when temperature-induced changes in quantitative MR quantities are computed between these tissue types. However, we note that there is consistently close agreement in the temperature readings (i.e., within 1 °C) from the rectal (body) thermometer, the temperature probes on the surface of the tumor, and MR thermometry. This supports the hypothesis that tumor temperatures were consistently comparable to the core body temperature (see Figure 3b). The limitations (iii) and (iv) may be addressed in future work to further validate the technique proposed in this preclinical pilot study. An orthotopic tumor model would also create a better testing environment to ensure that the observed changes can be properly linked to tumor biology.

In this work, we assume that the spin echo sequences produced accurate measurements of quantitative T_1_, T_2_, and ADC values. As with all in vivo imaging studies, there are potential sources of imaging-related errors that may adversely impact the accuracy and precision of these measurements. These issues include magnetic field inhomogeneity effects, imperfect RF pulses, slice profile, multi-slice collection, misregistration, partial volume effects/non-monoexponential relaxation, flow, diffusion, and magnetic susceptibility [37,38,39,40]. Attempts were made here to mitigate these effects. We carefully selected the RF transmit gain to produce correct 90/180 degree flip angles for our experiments. Non-perfect pulse calibration would tend to produce T_1_/T_2_ values systematically lower than expected [41]. For multislice collections, we used a non-contiguous acquisition to mitigate off-resonance pulse effects [42]. Signal intensity in a fitted region may be a mixture of various components and is also dependent on proton exchange rates. This means that each fitted region may have a different temperature sensitivity. In all cases, our residual values were small compared to the x–y values in the fits (see example in Figure 2c). In addition, the standard deviation of quantitative T_1_ measurements was low for multiple regions selected across tumor or muscle tissue. Therefore, we determined that (i) using a mono-exponential fit is a good approximation to accurately represent the individual fitted region spin-systems, and (ii) that the tissue was heated uniformly within this experimental setup. A similar approach was used to measure T_2_ and ADC.

## 5. Conclusions

In conclusion, we presented results that support the feasibility of a novel MR thermal contrast biomarker for tumor characterization. Future work is needed to (i) validate the technique in a larger cohort of preclinical subjects prior to clinical studies and (ii) combine this technique with other MR imaging methods in a multiparametric framework. Overall, the proposed thermal contrast may add new information to improve tumor characterization, which can improve cancer diagnosis and real-time MR-guided treatment monitoring.

## 6. Patents

Tissue delineation and characterization in magnetic resonance imaging. Matthew Richard Tarasek, Thomas Kwok-Fah Foo, Desmond Teck Beng Yeo, Oguz Akin. Patent number: 10,405,773.

## Figures and Tables

**Table 1:** Summary of (i) quantitative quantities ^a^ and (ii) thermally-induced changes in quantitative quantities ^b^. Bolded quantities indicate t-test *p*-value < 0.05 for comparisons indicated in text.

	Fibroadenoma (Benign)		Breast Tumor/MAT B III		Prostate Tumor/ MatLyLuB2	
	Muscle ^c^	Benign Tumor	Muscle ^c^	Breast Malignant	Muscle ^c^	Prostate Malignant
T_1_ (ms) ^a^	769 ± 12	651 ± 20	767 ± 10	1201 ± 26	770 ± 13	1145 ± 22
%ΔT_1_/°C ^b^	1.16 ± 0.20	1.20 ± 0.91	1.40 ± 0.42	**3.41** ± 0.64	1.05 ± 0.3	0.60 ± 0.51
T_2_ (ms) ^a^	26.0 ± 0.75	23.8 ± 1.2	25.8 ± 0.8	63.7 ± 2.1	26.6 ± 0.6	64.6 ± 1.7
%ΔT_2_/°C ^b^	0.50 ± 0.3	1.17 ± 0.8	0.72 ± 0.5	1.11 ± 0.7	0.70 ± 0.3	**1.7** ± 0.9
ADC (×10^−4^ mm^2^/s) ^a^	14.88 ± 1.1	11.22 ± 1.6	14.91 ± 1.0	8.9 ± 1.7	13.52 ± 1.2	10.3 ± 1.4
%ΔADC/°C ^b^	1.6 ± 0.7	**4.00** ± 0.78	1.8 ± 0.6	**4.20** ± 0.81	1.5 ± 0.6	**2.60** ± 0.56

^a^ Measurements were made at ~36 °C. Error reported from the calculated measurement stdev, ^b^ Measurements calculated as percent increase per degree Celsius. Absolute error reported from the propagation of 90% confidence intervals calculated as Ᾱ ± (*ts*/√*n*) where Ᾱ is the mean measured value, *t* is Student’s *t*, *s* is the measured stdev, and *n* is the number of measurements, ^c^ Muscle ROIs were selected near the corresponding benign or malignant tumors.

## Data Availability

The data presented in this study are available on request from the corresponding author. The data are not publicly available due to privacy.

## References

[1] Beck E.S., Gai N., Filippini S., Maranzano J., Nair G., Reich D.S. (2020). Inversion recovery susceptibility weighted imaging with enhanced T2 weighting at 3 T improves visualization of subpial cortical multiple sclerosis lesions. Investig. Radiol..

[2] Gillies R.J., Raghunand N., Karczmar G.S., Bhujwalla Z.M. (2002). MRI of the tumor microenvironment. J. Magn. Reson. Imaging Off. J. Int. Soc. Magn. Reson. Med..

[3] Joseph C.R. (2020). Novel MRI techniques identifying vascular leak and paravascular flow reduction in early Alzheimer disease. Biomedicines.

[4] Wu J.S., Hochman M.G. (2009). Soft-tissue tumors and tumorlike lesions: A systematic imaging approach. Radiology.

[5] Hakumäki J.M., Gröhn O.H., Tyynelä K., Valonen P., Ylä-Herttuala S., Kauppinen R.A. (2002). Early gene therapy–induced apoptotic response in BT4C gliomas by magnetic resonance relaxation contrast T 1 in the rotating frame. Cancer Gene Ther..

[6] Mountford C.E., Doran S., Lean C.L., Russell P. (2004). Proton MRS can determine the pathology of human cancers with a high level of accuracy. Chem. Rev..

[7] Simonetti A.W., Melssen W.J., Edelenyi F.S.d., van Asten J.J., Heerschap A., Buydens L.M. (2005). Combination of feature-reduced MR spectroscopic and MR imaging data for improved brain tumor classification. NMR Biomed. Int. J. Devoted Dev. Appl. Magn. Reson. Vivo.

[8] Choyke P.L., Dwyer A.J., Knopp M.V. (2003). Functional tumor imaging with dynamic contrast-enhanced magnetic resonance imaging. J. Magn. Reson. Imaging Off. J. Int. Soc. Magn. Reson. Med..

[9] Knopp M., Hoffmann U., Brix G., Hawighorst H., Junkermann H., van Kaick G. (1995). Fast MRI contrast medium dynamics for characterization of tumors. Experiences with functional MR-mammography. Der Radiol..

[10] Ma S., Nguyen C.T., Han F., Wang N., Deng Z., Binesh N., Moser F.G., Christodoulou A.G., Li D. (2020). Three-dimensional simultaneous brain T1, T2, and ADC mapping with MR Multitasking. Magn. Reson. Med..

[11] Méndez C.A., Pizzorni Ferrarese F., Summers P., Petralia G., Menegaz G. (2012). DCE-MRI and DWI integration for breast lesions assessment and heterogeneity quantification. Int. J. Biomed. Imaging.

[12] Damadian R., Zaner K., Hor D., DiMaio T. (1974). Human tumors detected by nuclear magnetic resonance. Proc. Natl. Acad. Sci. USA.

[13] Bloembergen N., Purcell E., Pound R. (1947). Nuclear magnetic relaxation. Nature.

[14] Chenevert T.L., Galbán C.J., Ivancevic M.K., Rohrer S.E., Londy F.J., Kwee T.C., Meyer C.R., Johnson T.D., Rehemtulla A., Ross B.D. (2011). Diffusion coefficient measurement using a temperature-controlled fluid for quality control in multicenter studies. J. Magn. Reson. Imaging.

[15] Parker D.L. (1984). Applications of NMR imaging in hyperthermia: An evaluation of the potential for localized tissue heating and noninvasive temperature monitoring. IEEE Trans. Biomed. Eng..

[16] Rieke V. (2011). MR thermometry. Interventional Magnetic Resonance Imaging.

[17] Graham S., Stanisz G., Kecojevic A., Bronskill M., Henkelman R. (1999). Analysis of changes in MR properties of tissues after heat treatment. Magn. Reson. Med. Off. J. Int. Soc. Magn. Reson. Med..

[18] Graham S.J., Bronskill M.J., Henkelman R.M. (1998). Time and temperature dependence of MR parameters during thermal coagulation of ex vivo rabbit muscle. Magn. Reson. Med..

[19] Han M., Rieke V., Scott S.J., Ozhinsky E., Salgaonkar V.A., Jones P.D., Larson P.E., Diederich C.J., Krug R. (2015). Quantifying temperature-dependent T 1 changes in cortical bone using ultrashort echo-time MRI. Magn. Reson. Med..

[20] Vesanen P.T., Zevenhoven K.C., Nieminen J.O., Dabek J., Parkkonen L.T., Ilmoniemi R.J. (2013). Temperature dependence of relaxation times and temperature mapping in ultra-low-field MRI. J. Magn. Reson..

[21] Parmala M., Eriksson M., Rytioja M., Tanttu J., Köhler M. (2016). Temperature measurement in human fat with T2 imaging. J. Magn. Reson. Imaging.

[22] Kuroda K., Oshio K., Chung A.H., Hynynen K., Jolesz F.A. (1997). Temperature mapping using the water proton chemical shift: A chemical shift selective phase mapping method. Magn. Reson. Med..

[23] Shmatukha A.V., Harvey P.R., Bakker C.J. (2007). Correction of proton resonance frequency shift temperature maps for magnetic field disturbances using fat signal. J. Magn. Reson. Imaging Off. J. Int. Soc. Magn. Reson. Med..

[24] Soher B.J., Wyatt C., Reeder S.B., MacFall J.R. (2010). Noninvasive temperature mapping with MRI using chemical shift water-fat separation. Magn. Reson. Med..

[25] Wyatt C.R., Soher B.J., MacFall J.R. (2010). Correction of breathing-induced errors in magnetic resonance thermometry of hyperthermia using multiecho field fitting techniques. Med. Phys..

[26] Hofstetter L.W., Yeo D.T., Dixon W.T., Kempf J.G., Davis C.E., Foo T.K. (2012). Fat-referenced MR thermometry in the breast and prostate using IDEAL. J. Magn. Reson. Imaging.

[27] Tarasek M.R., Pellicer R., Hofstetter L.W., Numan W.C., Bakker J.F., Kotek G., Togni P., Verhaart R.F., Fiveland E.W., Houston G.C. (2014). Validation of MR thermometry: Method for temperature probe sensor registration accuracy in head and neck phantoms. Int. J. Hyperth..

[28] MacFall J.R., Wehrli F.W., Breger R.K., Johnson G.A. (1987). Methodology for the measurement and analysis of relaxation times in proton imaging. Magn. Reson. Imaging.

[29] de Marini P., Cazzato R.L., Garnon J., Shaygi B., Koch G., Auloge P., Tricard T., Lang H., Gangi A. (2019). Percutaneous MR-guided prostate cancer cryoablation technical updates and literature review. BJR Open.

[30] Gangi A., Tsoumakidou G., Abdelli O., Buy X., de Mathelin M., Jacqmin D., Lang H. (2012). Percutaneous MR-guided cryoablation of prostate cancer: Initial experience. Eur. Radiol..

[31] Hua Y., Ma S., Fu Z., Hu Q., Wang L., Piao Y. (2011). Intracavity hyperthermia in nasopharyngeal cancer: A phase III clinical study. Int. J. Hyperth..

[32] Huilgol N.G., Gupta S., Sridhar C. (2010). Hyperthermia with radiation in the treatment of locally advanced head and neck cancer: A report of randomized trial. J. Cancer Res. Ther..

[33] Hynynen K., Pomeroy O., Smith D.N., Huber P.E., McDannold N.J., Kettenbach J., Baum J., Singer S., Jolesz F.A. (2001). MR imaging-guided focused ultrasound surgery of fibroadenomas in the breast: A feasibility study. Radiology.

[34] Mahnken A.H., Günther R.W., Tacke J. (2004). Radiofrequency ablation of renal tumors. Eur. Radiol..

[35] Caravan P., Ellison J.J., McMurry T.J., Lauffer R.B. (1999). Gadolinium (III) chelates as MRI contrast agents: Structure, dynamics, and applications. Chem. Rev..

[36] Golman K., Olsson L.E., Axelsson O., Mansson S., Karlsson M., Petersson J. (2003). Molecular imaging using hyperpolarized 13C. Br. J. Radiol..

[37] Bottomley P.A., Hardy C., Argersinger R., Allen-Moore G. (1987). A review of ^1^H nuclear magnetic resonance relaxation in pathology: Are T_1_ and T_2_ diagnostic?. Med. Phys..

[38] Just M., Higer H., Pfannenstiel P. (1988). Errors in T1-determination using multislice technique and Gaussian slice profiles. Magn. Reson. Imaging.

[39] Katz J., Boxt L.M., Sciacca R.R., Cannon P.J. (1990). Motion dependence of myocardial transverse relaxation time in magnetic resonance imaging. Magn. Reson. Imaging.

[40] Majumdar S., Sostman H.D., MacFall J. (1989). Contrast and accuracy of relaxation time measurements in acquired and synthesized multislice magnetic resonance images. Investig. Radiol..

[41] Bottomley P.A., Andrew E.R. (1978). RF magnetic field penetration, phase shift and power dissipation in biological tissue: Implications for NMR imaging. Phys. Med. Biol..

[42] Dixon W.T., Engels H., Castillo M., Sardashti M. (1990). Incidental magnetization transfer contrast in standard multislice imaging. Magn. Reson. Imaging.

